# The Treatment of Psychotic and Bipolar Disorders Within the South African Context: Perspectives of a Clinical Pharmacist

**DOI:** 10.3390/healthcare13121456

**Published:** 2025-06-17

**Authors:** Kudzai D. Kahwenga, Lindiwe Mnukwa, Elmien Bronkhorst

**Affiliations:** 1 School of Pharmacy, Department of Clinical Pharmacy, Sefako Makgatho Health Sciences University, Ga-Rankuwa 0208, South Africa; mnukwa.lindiwe@gmail.com (L.M.); elmien.bronkhorst@smu.ac.za (E.B.); 2 Department of Pharmacy, Dr George Mukhari Academic Hospital, Ga-Rankuwa 0208, South Africa

**Keywords:** clinical pharmacists, psychopharmacology, treatment guidelines, psychotic disorders, bipolar disorders, medication-related problems, South Africa

## Abstract

**Background:** The effective management of psychotic and bipolar disorders in tertiary care can improve patient outcomes, yet the role of clinical pharmacists in optimising psychotropic medication use remains underexplored in South Africa. This study aims to investigate the role and interventions of clinical pharmacists in managing psychotic and bipolar disorders within a tertiary hospital in South Africa. **Methods:** A quantitative, descriptive study was conducted among 60 adult patients admitted to the psychiatric and internal medicine wards diagnosed with psychotic and/or bipolar disorder. A previously validated, standardised pharmaceutical care form was utilised for a purposive sample of inpatient files. Medication-related problems were identified, and appropriate interventions were suggested. Prescriptions were also assessed for adherence to treatment guidelines, including the South African Standard Treatment Guidelines, the American Psychiatric Association guidelines, and the National Institute for Health and Care Excellence guidelines. **Results:** The study included 60 patients (37 females) with a mean age of 37 years. Diagnoses included schizophrenia (28.8%), bipolar disorder (27.5%), and stimulant-induced psychosis (19.3%). Sixty-two medication-related problems were identified, leading to 77 proposed interventions, of which 65 were implemented. Among the prescriptions, 75% (*n* = 45) adhered to the South African Standard Treatment guidelines, 76% (*n* = 46) adhered to the NICE guidelines, and 71% (*n* = 43) adhered to the APA guidelines. **Conclusions:** Clinical pharmacists identified a number of medication-related problems in patients with psychotic and bipolar disorders, and their proposed interventions were largely accepted. The findings highlight the pharmacist’s role in optimising medication therapy and adherence to guidelines, suggesting that improved treatment monitoring is necessary in this setting.

## 1. Introduction

Psychotic and bipolar disorders are two psychiatric conditions that significantly impact the well-being and daily life of those affected. These disorders often display overlapping clinical features, making accurate diagnosis and appropriate treatment crucial [1]. Bipolar disorder, previously known as manic-depressive disorder, is a chronic mood disorder characterised by a combination of elevated moods, hypomanic and depressive episodes, with accompanying sub-syndromal symptoms [2,3]. In contrast, psychotic disorders are severe mental illnesses marked by delusions, hallucinations, thought disturbances, and abnormal perceptions and behaviours [4,5]. To aid healthcare providers and patients in making informed decisions, treatment guidelines play a pivotal role in managing these disorders [6]. These guidelines not only enhance the consistency of care but also provide authoritative recommendations that bridge the gap between healthcare policies, best practices, local contexts and individual patient preferences [7].

Psychotic disorders often require the use of first- and second-generation antipsychotics, mood stabilisers, benzodiazepines and/or anticonvulsants, while bipolar disorder treatment typically involves mood stabilisers, anticonvulsants and/or antipsychotics [8]. These medications are not used simultaneously but are prescribed based on the specific needs and symptoms of the patient [8,9]. Occasionally, anticholinergics and beta-blockers are added for the management of antipsychotic-related side effects [8,10]. In prescribing these psychotropic medications, a comprehensive evaluation considering factors like the nature of the clinical condition, the risk–benefit profile of the medication and inherent pharmacological properties is essential [11]. Additionally, comorbidities, cost-effectiveness and the ongoing monitoring of treatment outcomes are integral concerns [11].

Within the realm of mental healthcare, pharmacists working in psychiatric settings often play a crucial role in medication management and patient education to enhance adherence to psychotropic medications. However, for optimal psychiatric treatment, clinical pharmacists are indispensable in leveraging their expertise in psychopharmacology and advanced clinical training [12]. Clinical pharmacists are able to combine a patient’s current diagnosis, laboratory results, medical history, and treatment guidelines with their knowledge of pharmacotherapy to identify any potential therapeutic errors or adverse drug events [13]. This holistic approach ensures that patients receive the most effective and safest treatment tailored to their unique needs, ultimately improving their overall quality of life.

Over the past few years, the clinical pharmacy profession in South Africa has experienced substantial growth and development. This transformation has shifted the focus from a product-oriented approach to a patient-oriented one, elevating the significance of clinical pharmacists in healthcare [14].

In South Africa, clinical pharmacists utilise a set of pharmaceutical care medication-evaluation interventions based on the guidelines established by the American Society of Hospital Pharmacists [14]. These interventions include various forms, such as the Pharmacist’s Patient Database Form, Current Drug Therapy Form, Laboratory Information, Drug Therapy Problem List, Drug Therapy Assessment Worksheet, and Pharmacist’s Care Plan Monitoring Worksheet. These care forms aim to optimise the use of medications, reduce potential drug interactions and side effects, and closely monitor therapeutic outcomes [14]. The types of interventions that can be made by the clinical pharmacist are shown in Table 1.

Studies have been published about clinical pharmacy services in several settings, including critical care [15,16], geriatrics [17], and ambulatory care, but very few have focused specifically on clinical pharmacist interventions in the inpatient setting of a psychiatry unit, especially in South Africa [12].

This study aimed to assess the contribution of clinical pharmacists in the treatment of psychotic and bipolar disorders at a tertiary hospital in South Africa and provide an overview of the potential interventions used to reduce the incidence of medication-related problems in this patient population.

## 2. Methods and Materials

A quantitative, descriptive research design was utilised in the study, where prospective data were collected over a period of three months, between November 2022 and February 2023, excluding Christmas holidays, using the standardised pharmaceutical care form.

### 2.1. Research Setting

The research setting was an academic hospital in South Africa. The psychiatry ward and internal medicine unit had 44 and 32 beds, respectively. Data collection spanned over three months, commencing in November 2022 and concluding in February 2023.

### 2.2. Inclusion/Exclusion Criteria

The inclusion criteria for the study were inpatient files of individuals aged over 18 years who had been diagnosed with psychotic and/or bipolar disorders and who were admitted to either the psychiatric or internal medicine wards. The exclusion criteria included patient files of minors younger than 18 years, patients with diagnoses not including psychotic or bipolar disorders, and those admitted to non-medical wards during the study period.

### 2.3. Participants

A non-probability purposive sampling method was used for the sample selection. This involved the researcher, a clinical pharmacist with expertise in the treatment of psychiatric disorders, prospectively selecting the inpatient files of adult patients who met the inclusion criteria (diagnosed with psychotic and/or bipolar disorders and admitted to psychiatric or internal medicine wards) as they became available during the three-month data collection period. This method was chosen to ensure that the selected cases were relevant to the study’s aim of investigating the clinical pharmacist’s role in managing these specific disorders and identifying associated medication-related problems. The required sample size (n) when conducting the study at the 95% confidence interval was 52 while using the 5% margin of error and a 50% response distribution. A sample size of *n* = 60 was included and was calculated using Raosoft^®^ Inc., Seattle, WA, USA.

### 2.4. Data Collection Instrument and Process

The data collection instrument used in the study was a previously validated form used in numerous pharmaceutical care studies, facilitating the consistent documentation of all clinical interventions. The pharmaceutical care form provided a structured process that assisted clinical pharmacists in delivering quality care to all patients. This process involved assessing the patient’s needs, developing a care plan, and subsequently monitoring the plan [14]. The pharmaceutical care form comprised four sections (see Appendix A.1):

- Section A: Patient Database. This includes demographic information, current and past medical history, and behavioural/lifestyle information. The patient was assigned a study number in this section.
- Section B: Current Medication List. The aim was to assess the prescribed medication and compare it to what the guidelines on the treatment of the condition said, with the intent of optimising the patient’s pharmacotherapy.
- Section C: Medication Therapy Assessment. This included assessing any medication-related problems with the use of guided questions that the researcher should review every day and use to monitor the patient.
- Section D: This section included the implemented interventions and evaluated the outcomes of those interventions.

The data collection process followed a stepwise approach: eligible patients were chosen based on the inclusion criteria. Their medical records were reviewed to compare the prescribed medications with treatment guidelines. Any issues related to medication were identified, discussed, intervened, and monitored. The findings and interventions were captured and analysed.

### 2.5. Data Analysis

The data were captured using Microsoft Excel® for Microsoft 365 MSO (Version 2505 Build 16.0.18827.20102) (Microsoft Corporation, Redmond, WA, USA) Microsoft Excel™. Analysis was performed descriptively using the Statistical Package for the Social Sciences (SPSS version 29) IBM® SPSS® Statistics for Windows, Version 29.0 (IBM Corp., Armonk, NY, USA) software with the assistance of a statistician. The demographic data were presented in terms of percentages, and graphical representation was used to examine guideline adherence, medication-related difficulties, and recommended interventions. Medication-related challenges and interventions were categorised and discussed systematically. If extra information was needed on the patient’s condition and current medication for clarity, the prescriber, not the patient, was consulted.

### 2.6. Ethical Considerations

An ethical clearance certificate to perform the study was obtained from the Institutional Research and Ethics Committee on the 4th of August 2022 (SMUREC/P/222/2022: PG.) Patient consent was waived due to the nature of the study; there was no direct interaction with the patients. Furthermore, permission was obtained from the Chief Executive Officer of the Tertiary Academic Hospital, the hospital superintendent, and responsible prescribers.

## 3. Results

### 3.1. Demographic and Health Characteristics of Study Participants

A total of sixty (*n* = 60) patients were included in the study over a period of three months. Among the included participants, 37 (61.7%; *n* = 60) were female with a mean age of 37 years, ranging from 19 to 71 years. The demographic and health characteristics of the participants are summarised in Table 2.

### 3.2. Comorbidities

This study examined the presence of comorbidities among selected patients (*n* = 60). Table 2 provides an overview of the most prevalent comorbidities identified. The most prevalent comorbidity was hypertension (nine patients; 15%), followed by HIV (five patients; 8.3%), type 2 diabetes mellitus (two patients; 3.3%), and epilepsy (two patients; 3.3%).

New comorbidities emerging after therapy initiation were observed. Two patients (one with schizophrenia and the other with bipolar 1 disorder) developed hypertension two months after therapy initiation, while another patient (with bipolar 1 disorder) developed diabetes; notably, all three patients were on risperidone.

### 3.3. Prevalence of Psychiatric Disorders

**Figure 1:**
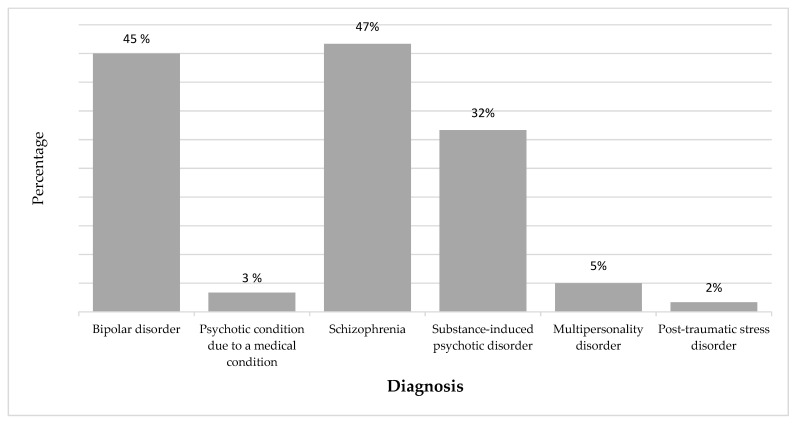
Diagnosis of psychotic and bipolar disorders.

Most of the patients, 47% (28), were diagnosed with schizophrenia. This diagnosis was found either alone or accompanied by other psychotic and/or bipolar disorders. Bipolar disorder was prevalent in 45% (27) of the patients seen, indicating a significant presence of this disorder within the study population. Substance-induced psychotic disorders were observed in 32% (19) of the patients (with the most abused substances being cannabis, alcohol, and crystal methamphetamine). Figure 1 shows the prevalence of these disorders.

### 3.4. Adherence to Treatment Guidelines

The adherence to treatment guidelines was evaluated by analysing the prescriptions written for the study participants. The findings regarding adherence to different guidelines are presented in Table 3. Of the prescriptions written, 75% (45; *n* = 60) adhered to the South African Standard Treatment (STG) guidelines, 76% (46; *n* = 60) adhered to the National Institute for Health and Care Excellence (NICE) guidelines, and 71% (43; *n* = 60) adhered to the American Psychiatric Association (APA) guidelines. Reasons for not adhering to the guidelines included systemic and medication supply barriers and the prescriber’s own alternative plans or assurance.

### 3.5. Medication-Related Challenges

**Figure 2:**
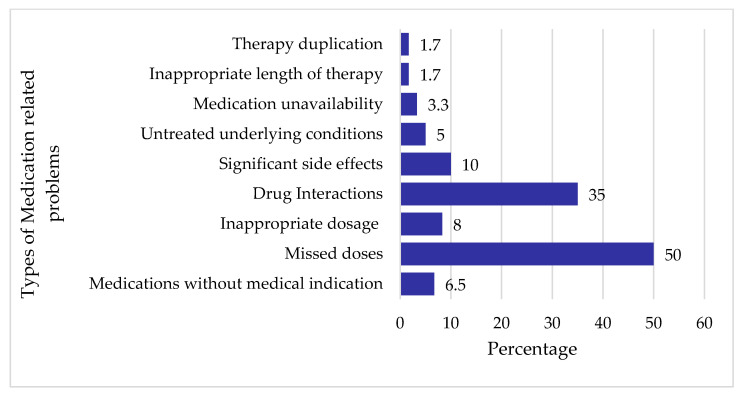
Medication-related problems.

A total of 62 medication-related problems, with an average of 1.03 problems per patient, were identified and documented. Figure 2 presents an overview of the most common medication-related problems observed in the study population.

#### 3.5.1. Missed Doses

One of the most frequently encountered medication-related problems was missed doses, accounting for 50% (N = 62) of the identified issues. This refers to instances where nurses fail to adhere to the prescribed medication regimen by omitting or forgetting to give the patients their scheduled doses.

#### 3.5.2. Drug Interactions

Another prevalent medication-related problem identified in this study was drug interactions, which accounted for 35% (22) of the total medication-related problems. Examples of major to moderate drug–drug interactions included the concomitant administration of risperidone with fluoxetine (used in the treatment of bipolar I disorder), risperidone with citalopram (for schizophrenia), and quetiapine with risperidone (for both schizophrenia and bipolar I disorder).”

Furthermore, drug–drug interactions were also identified when patients’ substance abuse interacted with their medication regimen.

#### 3.5.3. Inappropriate Dosage

Inappropriate dosages were identified as notable medication-related problems, comprising 8.06% (5) of the total problems.

#### 3.5.4. Medications Prescribed Without Indications

Medications prescribed without clear indications accounted for 6.45% (4) of the identified issues. This refers to situations where medications were prescribed without a documented diagnosis or a specific medical condition that warranted their use. This included tramadol at 50 mg, two patients on hydrochlorothiazide at 12.5 mg daily, and metformin at 500 mg twice daily.

### 3.6. Clinical Pharmacist-Led Interventions

A total of 77 interventions were proposed, out of which 65 interventions were accepted and successfully implemented; the number of interventions per patient varied and sometimes required more than one per patient, which is why there were more interventions than patients in the study.

Proposed interventions were delivered through three modes, as shown in Table 4. The total number of interventions made by consulting with nurses was 70% (54); those made through writing a prescription accounted for 3% (2) of the interventions; and 27% (21) of interventions were made by consulting with doctors (psychiatrists and medical doctors).

**Figure 3:**
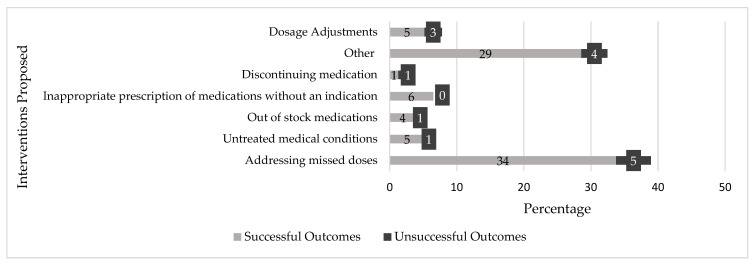
Clinical pharmacist-led interventions.

The most prevalent interventions, as shown in Figure 3, included addressing missed doses (34% successful outcomes of intervention, 5% unsuccessful outcomes of intervention), addressing untreated medical conditions (5% successful outcomes of intervention, 1% unsuccessful outcomes of intervention), and addressing the inappropriate prescription of medications without clear indications (6% successful). Reasons for unsuccessful outcomes included administrative and process delays, as well as communication gaps with nurses. Among prescribers, contributing factors included similar administrative delays and confidence in their original treatment plans. Lastly, a written communication that was either overlooked or not read also contributed.

These interventions aimed to optimise medication adherence, manage untreated medical conditions, and rectify inappropriate prescribing practices, respectively. The intervention process for addressing missed doses involved talking to nurses about the omission of doses in the prescription file and understanding the reasons behind it. Nurses were educated on the importance of administering medication at the prescribed frequency, and follow-up was conducted to ensure that the doses were being taken as prescribed.

Other interventions involved recommendations to be relayed to patients by nurses regarding social drug use and education, emphasising the importance of adherence to the prescribed medication regimen.

## 4. Discussion

This study evaluated the role and interventions of a clinical pharmacist in the management of psychotic and bipolar disorders in a South African tertiary hospital, hypothesising that their integration could enhance prescribing practices and medication safety. A three-month quantitative, descriptive study of 60 adult inpatients diagnosed with these disorders involved prospective data collection on medication-related problems (MRPs) using a standardised pharmaceutical care form. Pharmacist interventions, their acceptance, and adherence to treatment guidelines, including the South African Standard Treatment Guidelines (STGs), the National Institute for Health and Care Excellence (NICE) guidelines, and the American Psychiatric Association (APA) guidelines were documented. Schizophrenia and bipolar disorder were the predominant diagnoses. Key findings included 62 identified MRPs, including primarily missed doses and drug interactions; a high acceptance rate for pharmacist interventions; and varied adherence to treatment guidelines. This discussion will interpret these findings in the context of the existing literature, explore their implications, and consider the study’s limitations.

The study cohort, predominantly women with a mean age of 37, showed demographic and diagnostic profiles (schizophrenia, bipolar disorder, and substance-induced psychotic disorders) that partially align with existing research. Notably, unlike some South African and international studies reporting higher male admission rates [18,19,20,21], this study observed higher female admission and discharge rates. Ward staff attributed this discrepancy to challenges in post-discharge arrangements for male patients due to social worker intervention difficulties, leading to prolonged stays and reduced male admissions. This observation, though qualitative, suggests a systemic issue requiring policy attention to improve patient flow and equitable access to care. Regarding comorbidities, hypertension was most frequent, which is consistent with the literature indicating high rates of medical conditions like diabetes and cardiovascular diseases in patients with severe mental illness [22,23]. Bipolar disorder has also been reported to have a heightened prevalence of comorbid medical conditions, such as asthma, chronic bronchitis, and gastric ulcers [22,23].

The emergence of new comorbidities after the initiation of therapy occurred in three patients on risperidone, with two developing hypertension (one with schizophrenia and the other with bipolar 1 disorder) and one diagnosed with diabetes (diagnosed with bipolar 1 disorder). Previous studies have reported a correlation between the use of atypical antipsychotics and the risk of developing metabolic syndrome, which can subsequently lead to hypertension and diabetes [24,25]. It is important to acknowledge that several factors, such as family history, weight, and social drug use, contribute to the development of these comorbidities, making it crucial to approach the analysis of comorbidities in a comprehensive and multifactorial manner [26].

The majority of prescriptions adhered to all treatment guidelines. The most recent global survey on guideline development and methodological comparison took place in 2011 and was conducted by Gaebel et al. (2005). In the survey, both the APA and NICE guidelines were included, and the findings indicated that the NICE schizophrenia guidelines were unsurpassed in terms of methodological quality and practicality. Conversely, the APA guidelines excelled in presenting various options clearly and providing background information but were lacking in applicability [27]. In this study, deviations from treatment guidelines were attributed to various factors, including systemic and medication supply barriers—such as delays of the prescriber in medication renewal or the unavailability of the medication recommended in the guidelines. In some cases, prescribers opted not to follow the guidelines recommended due to clinical judgment or alternative care plans. South Africa’s public healthcare system lacks specific, comprehensive psychiatric management guidelines, with existing STGs being insufficient and the South African Society of Psychiatrists (SASOP) guidelines being primarily tailored for the private sector. Therefore, a combined approach that incorporates the recommendations of more than one guideline and additional research or expertise may provide a more comprehensive framework for guiding the management of psychotic and bipolar disorders [28].

Missed doses were a frequently encountered MRP. This finding, while from a smaller sample, focused on broader psychotic disorders compared to Bereda et al.’s (2021) Ethiopian study on schizophrenia [29], highlighting a persistent challenge. Non-compliance in inpatient psychiatry, estimated at between 20 and 80% [30], exacerbates relapse risks and inpatient costs and reduces quality of life [31].

In our research setting, while examining medication charts, we came across numerous empty administration signature boxes, and the rule of thumb is that “if it is not documented, then it was not done”. This highlights the role of the medication chart as a central communication tool among healthcare professionals, providing insight into the patient’s treatment journey, including instances of non-adherence, whether due to patient refusal, system failures, or other issues. Proper documentation could have offered greater clarity on the reasons for missed doses, which is particularly relevant in psychiatric settings where patient refusal is common [32]. Nursing administrators are key to fostering a culture of error reporting and prevention, enabling safer medication administration policies. Ward managers should encourage a positive view of error reporting among nurses, seeing it as a chance to comprehend error causes, analyse relationships, and establish effective policies for prevention [33].

Drug interactions were another prevalent medication-related problem identified. The major–moderate interactions identified (e.g., risperidone with sodium valproate, citalopram, or quetiapine) align with findings by Aburamadan et al. [34], and necessitate careful risk–benefit analysis for continued co-prescription [35].

Inappropriate dosages, including “as needed” (pro re nata-PRN) benzodiazepine and analgesic prescriptions lacking clear maximum daily doses, were also noted. This practice shifts dosing responsibility to nurses and risks over-sedation [36], indicating a need for stricter institutional prescribing policies for PRN medications, mandating clear indications and dosage parameters.

The off-label prescription of metformin, reportedly used for antipsychotic-induced weight management, reflects an area where clinical practice is ahead of, or divergent from, current guidelines [37,38,39]. While some studies support metformin’s efficacy for such metabolic issues [37], and emerging evidence suggests other neurological benefits [40], its formal inclusion in treatment guidelines requires rigorous evaluation, especially considering risks like lactic acidosis in patients with alcohol abuse—a relevant concern for this population [41].

The high acceptance rate (84.4%) of clinical pharmacist-led interventions observed in this study mirrors trends in other psychiatric settings [42,43,44].

The majority of the interventions were made by consulting with nurses since the presence of prescribers did not consistently align with the researcher’s presence. The most common interventions were addressing missed doses, untreated medical conditions, and addressing the inappropriate prescribing of medications without an indication. Other interventions included dose adjustments, recommendations to be relayed to the patients by nurses on social drug use and the importance of adherence and education. Interventions involving physicians primarily focused on clinical decision-making and medication safety. These included confirming medication scripts to ensure appropriate prescribing, alerting doctors to abnormal electrolyte levels and potential drug–drug interactions and discussing the initiation or adjustment of medications in patients with comorbidities. They were also advised to consider dose modifications in cases of excessive sedation from pain medication. In contrast, interventions involving nurses centred on patient education and adherence support. They were guided on the importance of administering medications at prescribed intervals and educating patients—especially those on antiretroviral therapy—on the necessity of consistent adherence.

These findings, similar to Stuhec et al. (2021) [43], underscore the pharmacist’s role in rectifying medication omissions, especially for chronic conditions. The reasons behind the unsuccessful outcomes of interventions primarily stemmed from administrative and process delays, such as interruptions in therapy due to delays in prescription renewals or prescriber reviews. Communication gaps included handover issues between nurses and prescribers during shifts. Additionally, in some cases, prescribers opted not to adopt recommendations based on their own clinical judgment or pre-existing alternative care plans when inquired by the clinical pharmacist. System-level improvements could be made, such as instituting clear, trackable protocols for submitting, reviewing, and responding to clinical pharmacy recommendations and embedding regular, dedicated interdisciplinary discussions about medication management into team workflows (e.g., during patient handovers or in multidisciplinary team rounds) to fully support and integrate this expertise for optimal, guideline-based patient care.

In this study, the clinical pharmacist’s interventions included medication reconciliation, the identification and resolution of drug-related problems, and the provision of patient-specific education to healthcare staff.

Based on this study’s findings, several recommendations are proposed. Firstly, there is a suggestion to enhance the dose administration system by implementing a thorough documentation process involving a two-step verification system with a second staff member available for accuracy validation. Secondly, allocating dedicated full-time clinical pharmacists to inpatient psychiatric wards is recommended for comprehensive daily medication management. Lastly, to improve collaboration between clinical pharmacists and prescribers, scheduled joint ward rounds or dedicated medication review meetings could be implemented. Showcasing the pharmacist’s impact through regular feedback on interventions and patient progress might help improve the collaboration between clinical pharmacists and prescribers.

### Strengths and Limitations of the Study

This study has several strengths. It prospectively collected data using a validated pharmaceutical care form, providing a systematic approach to identifying medication-related problems and pharmacist interventions in a real-world clinical setting. The focus on the under-researched area of clinical pharmacists’ impact on psychotic and bipolar disorder management in a South African tertiary hospital adds valuable regional data. Furthermore, the high acceptance rate of clinical pharmacist-led interventions underscores their perceived value and collaborative potential within the healthcare team.

This study also has limitations. The research was conducted in a single tertiary academic hospital, which may limit the generalisability of the findings to other types of healthcare settings or regions within South Africa. The sample size, while meeting calculated requirements for the 95% confidence interval, was relatively modest (n = 60), and a larger cohort might reveal further nuances. The data collection period was three months; a longer duration could account for seasonal variations or changes in staffing that might influence practices. The absence of a control or comparison group limited causal inference. The observed medication-related problems were not categorised according to their severity, which would have added more depth to the methodology and interventions made.

While prescribers were consulted for clarity, this study did not involve direct patient interaction to assess their perspectives on medication management, adherence, or clinical improvement. Additionally, the study noted challenges in post-discharge arrangements, particularly for male patients, which impacted admission rates but was an external factor to the core pharmaceutical interventions assessed.

## 5. Conclusions

This study highlights the need for improved treatment monitoring and intervention in the management of psychotic and bipolar disorders within a South African public tertiary hospital. The significant role of clinical pharmacists in identifying and resolving medication-related problems was evidenced by the high acceptance rate of their recommendations. These findings strongly support the integration of dedicated clinical pharmacy services into psychiatric care models. The integration of psychiatric stewardship into South Africa’s healthcare system could represent a substantial advancement, ensuring consistent, high-quality care for patients throughout their treatment. While this study underscores the value of clinical pharmacists, its findings are based on a single centre with a modest sample size over a three-month period, indicating the need for larger, multi-centre studies to confirm these benefits more broadly.

Further research should explore the long-term clinical and economic benefits of such integrated care models in similar settings.

## Figures and Tables

**Table 1:** Medication-related problems identifiable by a clinical pharmacist and associated outcome measures [14].

Therapeutic Problem	Explanation	Monitoring/Outcome Measure
Lack of correlation between drug therapy and medical problems: inappropriate drug selection	Drugs without medical indications, unidentified medications, or untreated medical conditions, including any which require drug therapy. Comparative efficacy, safety, and appropriateness for the individual patient.	Discontinue or introduce drugs. Destroy unidentified medication. Limit side effects or adverse drug reactions.
Drug regimen	Inappropriate dose, dosing frequency, dosage form, route of administration (considering efficacy, safety, and convenience), or duration of therapy.	Optimise dose to reduce adverse drug reactions. Optimise the dosing regimen, including dose frequency, form, and route of administration.
Therapeutic duplication	The treatment of any condition with more types of medication than necessary.	Optimise dose to reduce adverse drug reactions.
Drug allergy or intolerance	Any medicines and methods used to alert healthcare providers to the allergy/intolerance.	Avoid hypersensitivity reactions. Ensure healthcare providers are aware of allergy. Identify and stop offending medicine.
Adverse drug events	Any possibly drug-related symptoms or medical problems, and the likelihood that the problem is drug-related.	Report ADR on pharmacovigilance form Identify interactions and discontinue/replace the identified drugs. Reduce adverse drug reactions.
Interactions	Drug–drug interactions, drug–disease interactions, drug–nutrient interactions, and drug–laboratory interactions.	Report ADR on pharmacovigilance form. Identify interaction and discontinue/replace identified drug.
Social or recreational drug use	Smoking or alcohol. Recreational drugs.	Identify problems caused by social drug use.
Failure to receive therapy	Reasons such as system errors or any other factors that could hinder the achievement of therapeutic efficacy.	Recommend optimal management. Ensure the availability of medicine supplies to patients. Address other factors or system errors.

**Table 2:** Demographic and health characteristics of study participants.

Characteristics	Number	Percentage (%)
**Total patients (n)**	60	
**Gender:**		
Female	37	61.7
Male	23	38.3
**Age:**		
Mean (years)	37	-
Range (years)	19–71	-
**Comorbidities**	**Frequency**	**Percentage %**
Epilepsy	2	3.3
HIV positive	5	8.3
Hypertension	9	15
Peripheral vascular disease	1	1.7
Type 2 diabetes mellitus	2	3.3
No comorbidities	41	68.3

**Table 3:** Prescribers’ adherence to treatment guidelines.

	Compliance to NICE	Compliance to STG	Compliance to APA
Frequency	46	45	43
Percentage (%)	76	75	71

**Table 4:** Modes of clinical pharmacist interventions and outcomes.

Mode of Intervention	Sum of Frequency	Percentage (%)	Successful Outcomes from Interventions	Unsuccessful Outcomes from Interventions	Reason for Unsuccessful Outcome
Communication with the nurses	54	70	51	3	Administrative and process delays (2). Communication gaps (1).
Communication with the doctors	21	27	13	8	Doctor assurance of treatment plan (5). Administrative and process delays (3).
Written in the file communication	2	3	1	1	Communication not read (1).

## Data Availability

The data supporting the findings of this study are not publicly available due to ethical and privacy restrictions. The data are owned by Sefako Makgatho Health Sciences University and includes sensitive patient information that was anonymised and securely stored. Access was restricted to the researcher and supervisors only, in compliance with ethical and intellectual property requirements.

## Appendix A

### Appendix A.1. The Data Collection Instrument (Pharmaceutical Care Form) Used in This Study as Mentioned in Section 2.4 [14]

Data collection instrument (pharmaceutical care form).
